# Exosomes in Hepatocellular Carcinoma: A Comprehensive Review of Current Research and Future Directions

**DOI:** 10.1111/jcmm.70723

**Published:** 2025-07-27

**Authors:** Subham Sarkar, Ashok Kumar Bishoyi, R. Roopashree, Vishal Thakur, Manpreet Kaur, Pusparghya Pal, Taha Alqahtani, Ali Alqahtani, Daniel Ejim Uti, Esther Ugo Alum

**Affiliations:** ^1^ Center for Global Health Research, Saveetha Medical College and Hospital Saveetha Institute of Medical and Technical Sciences Chennai India; ^2^ Department of Microbiology, Faculty of Science Marwadi University Rajkot Gujarat India; ^3^ Department of Chemistry and Biochemistry, School of Sciences JAIN (Deemed to Be University) Bangalore Karnataka India; ^4^ Centre for Research Impact & Outcome, Chitkara University Institute of Engineering and Technology Chitkara University Rajpura Punjab India; ^5^ Department of Pharmacy, Chandigarh Pharmacy College Chandigarh Group of Colleges‐Jhanjeri Mohali Punjab India; ^6^ NIMS Institute of Pharmacy NIMS University Rajasthan Jaipur India; ^7^ Department of Pharmacology, College of Pharmacy King Khalid University Abha Saudi Arabia; ^8^ Department of Biochemistry/Research and Publications Kampala International University Kampala Uganda; ^9^ Department of Biochemistry, Faculty of Basic Medical Sciences, College of Medicine Federal University of Health Sciences Otukpo Benue State Nigeria

**Keywords:** clinical trials in HCC, exosome therapy, exosome‐based diagnostics, hepatocellular carcinoma, isolation techniques for exosomes, tumour diagnostics

## Abstract

Hepatocellular carcinoma (HCC) is a common form of liver cancer that is deadly and offers limited possible treatment options. This short review explored the role of exosomes (small vesicles released by cells) in HCC as either diagnostic or therapeutic possibilities. Exosomes facilitate tumour growth by carrying tumour‐supportive material to promote angiogenesis and metastasis. At the same time, exosomes may serve as a tumour biomarker for early diagnosis or prognostic possibilities in HCC. In the future, exosomes may be used as targeted therapies for HCC patients by enabling engineered exosomes to deliver therapeutics to tumour cells with more specificity and lower side effects. Lastly, this review discussed exosome isolation and characterisation techniques, engineering engineered exosomes for therapeutics and clinical trials using exosomes as HCC treatment options. Many challenges remain, including scale of production and standardisation aspects; the future development of exosome therapeutics appears promising. This review underscores the importance of continuing research towards improving exosome technologies and using exosomes in combination therapies, as they may help develop safer and more efficient ways to improve HCC care.

## 1 Introduction

**FIGURE 1:**
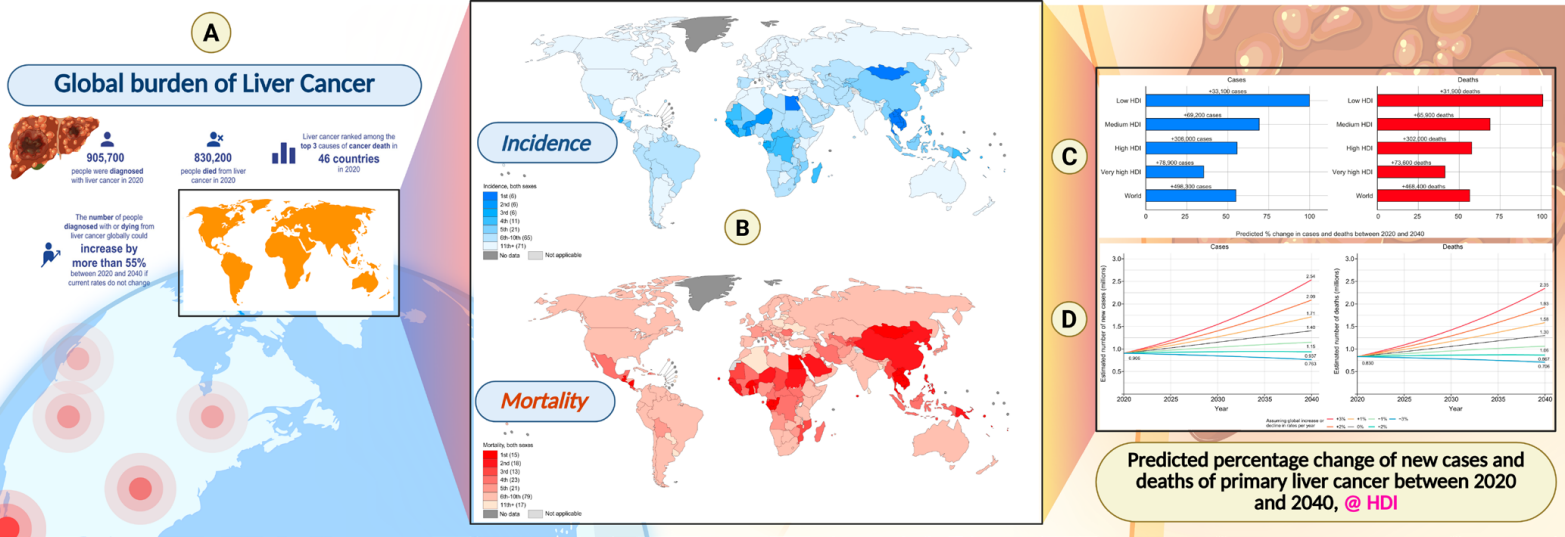
(A) Global burden of primary liver cancer in 2020 and predictions to 2040; (B) ranking of primary liver cancer among other cancer types based on number of cases or deaths in 2020, by country; (C) predicted percentage change of new cases and deaths from primary liver cancer between 2020 and 2040, by HDI; (D) predicted number of new cases and deaths from primary liver cancer assuming seven scenarios of annual change in global rates between 2020 and 2040 (Reproduced with permission under Creative Commons CC BY 4.0 licence from ref. [3] Copyright 2022 The Authors).

HCC is the most common type of primary liver cancer, commonly seen in people with chronic liver conditions and cirrhosis. HCC is the third most common reason for cancer‐related death globally, with rising incidence across numerous areas [1]. HCC can occur from many different sources, including viral disease (hepatitis B and C), overdose of metabolic toxins (alcohol and aflatoxins) and nonalcoholic fatty liver disease and diabetes [2]. Figure 1 provides a detailed visualisation of the global burden of primary liver cancer and illustrates incidence and mortality rates throughout regions. In 2020, there were an estimated 905,700 new liver cancer cases and an estimated 830,200 deaths. The figure shows that liver cancer continues to be one of the top three cancer death causes in 46 countries. It also shows that cases and deaths are expected to increase by more than 55% by 2040 if the current trajectory continues. The maps show incidence and mortality by country, with darker colours indicating higher rates. The figure also includes bar graphs and line plots showing the predicted changes in liver cancer cases and deaths by Human Development Index (HDI), which indicates varied impacts depending on a country's socioeconomic context [3].

The treatment landscape for HCC is complex, owing to its frequent late‐stage diagnosis. Treatment modalities are stratified according to stage and include surgical resection and liver transplant, as well as ablation for early‐stage disease. For more advanced stages, options include systemic chemotherapy and targeted therapies [4]. Even with these treatment options, recurrence rates remain high and overall survival rates are low, indicating an urgent need for better treatment strategies and improved early detection methods [5].

**FIGURE 2:**
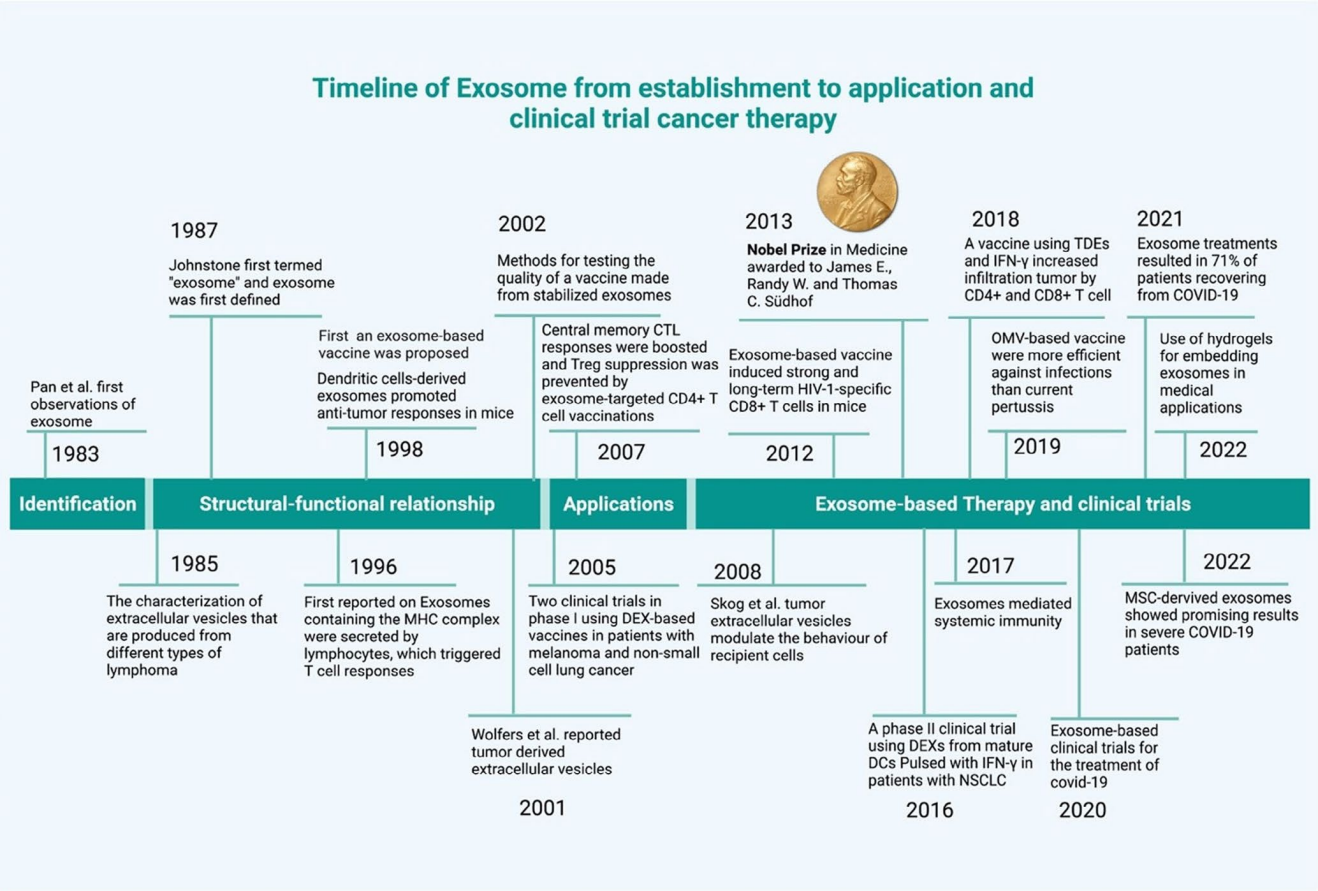
Exosomes‐based cancer therapy timeline (Reproduced with permission under Creative Commons CC BY 4.0 licence from ref. [8] Copyright 2022 The Authors).

Exosomes are small extracellular vesicles ranging from 30 to 150 nm that arise from the endosomal cellular system and serve the major function of enabling cellular communication through the transfer of proteins, lipids and nucleic acids. In cancer, exosomes are directly involved in tumour biology through alterations of the tumour microenvironment and evidence for promoting tumour cell migration as well as immune modulation [6]. Exosomes in HCC can have a dual role. They can promote tumour growth through a variety of mechanisms (including angiogenesis) while providing opportunities for targeted delivery of therapeutics. Because of their ability to carry and protect therapeutic agents (such as RNAi molecules or chemotherapy drugs), exosomes may represent a viable approach to counteract the drug resistance characteristic of HCC treatment. In addition, exosomes can be utilised to deliver tumour antigens to produce vaccines and immunotherapeutics [7]. Recent advances in microfluidics and the engineering of nanomaterials have produced exosome‐based chipsets that can isolate and characterise HCC‐specific exosomes in biological fluids (Figure 2). These devices also exploit molecular signatures (specific miRNA's and protein markers) to achieve noninvasive and early detection of HCC [9]. This technology holds promise for improving disease prognosis by enabling earlier and potentially more effective treatment interventions.

This review aims to consolidate current research on the role of exosomes in HCC, focusing on both their therapeutic and diagnostic capabilities. The unique aspect of this review is its comprehensive examination of exosome utilisation throughout HCC management, from early detection to innovative treatment approaches via thorough assessment of the present research landscape, identifying existing knowledge gaps and proposing future directions for research and clinical trials [10].

## 2 Current Research and Exosomes as Pioneering Frontiers in HCC

**FIGURE 3:**
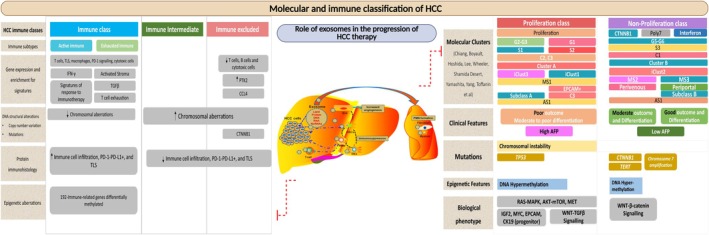
Molecular and Immune Classification of HCC and Role of Exosomes in Progression of HCC (Reproduced with permission under Creative Commons CC BY 4.0 licence from ref. [4] Copyright 2021, Reproduced with permission under Creative Commons CC BY 4.0 licence from ref. [13] Copyright 2023 The Authors).

In the complex and evolving landscape of HCC research, scientists strive to decode the intricate molecular and immune profiles that underpin this aggressive form of liver cancer. Recognising the diversity in tumour biology, researchers have segmented HCC into various subtypes based on genetic, metabolic and immune characteristics [11] This classification is significant in creating individualised therapies that are closely matched to the disease mechanisms in every individual. However, despite these advances, the considerable heterogeneity of HCC can outpace current classification systems, sometimes putting the clinician behind the eight ball when it comes to personalised care. At the same time, clinical/translational researchers have been developing a multitude of preclinical and clinical models to try to provide clinical utility to laboratory work. The research community is designing and optimising a host of in vitro and in vivo models, including both cell lines and organoids, and rodent models, to study the molecular pathways associated with HCC. These models are instrumental for testing new therapies and dissecting the disease at a molecular level. Yet, the journey from bench to bedside is fraught with challenges, as many models fail to fully replicate the human condition, particularly the complex tumour microenvironment, which is critical for understanding HCC's true nature. The treatment regime for HCC is as diverse as the disease itself, involving surgical resections, liver transplants and an array of systemic therapies tailored to disease staging. The treatments for early‐stage HCC are quite promising, yet the landscape for advanced HCC is starkly different, plagued by high rates of drug resistance and limited treatment efficacy [12]. This ugly reality underscores an urgent need for novel therapeutic strategies (Figure 3A–C).

Into this milieu of medical need, exosomes emerge as novel players with the potential to revolutionise HCC therapy. These nano‐sized extracellular vesicles naturally facilitate cellular communication and can be harnessed to deliver therapeutic agents directly into tumour cells. This innovative approach holds promise for surmounting the perennial challenge of drug resistance in advanced HCC. Beyond drug delivery, exosomes also offer prospects for immune modulation and vaccine development, opening new avenues for immunotherapy in HCC management. The exploration of exosomes in HCC treatment represents a hopeful frontier in the battle against liver cancer. Their unique capabilities in drug delivery and immune system engagement offer a dual approach to tackle both the disease's progression and the body's response to tumours [11, 13] (Figure 3D). This narrative continues to unfold as researchers diligently work to translate the therapeutic potential of exosomes into clinical realities, potentially setting the stage for a new era in the management of hepatocellular carcinoma.

## 3 Landscape of Exosome Biogenesis and Therapeutics in HCC

**FIGURE 4:**
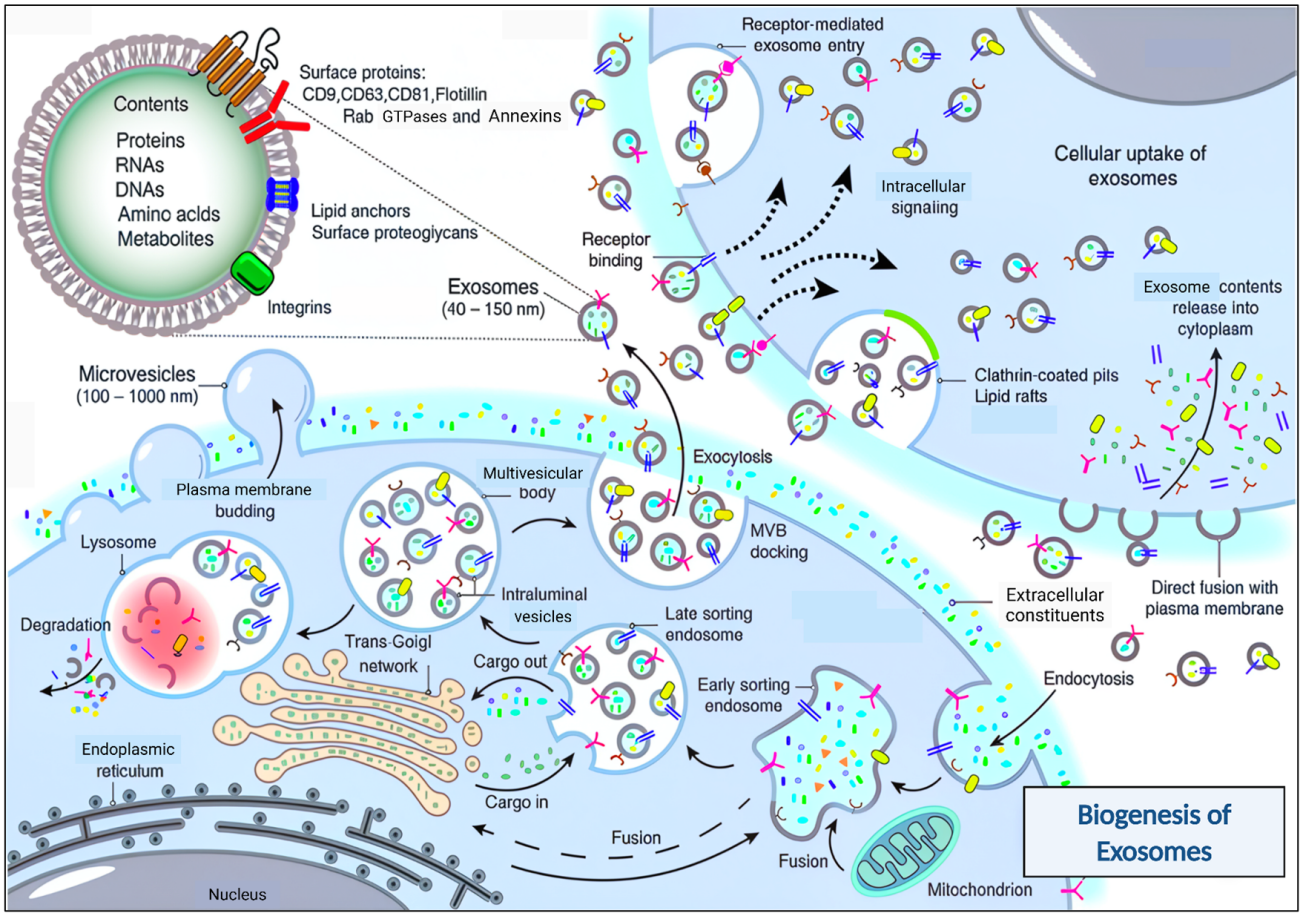
Exosome biogenesis (Reproduced with permission under Creative Commons CC BY 4.0 licence from ref. [14] Copyright 2024 The Authors).

Exosome biogenesis is a complex process integral to cellular communication and molecular biology. Exosomes, which are derived from the endosomal system within the cell, are formed in the endosomal compartment called multivesicular bodies (MVBs). MVBs have intraluminal vesicles (ILVs) that contain proteins, lipids and nucleic acids. When MVBs fuse with the plasma membrane, ILVs are secreted as exosomes (Figure 4). Exosomes can communicate with target cells through endocytosis and deliver their contents, influencing recipient cell function and behaviour [15, 16].

In the context of HCC, exosomes have novel characteristics which represent exciting opportunities for therapy. Exosomes can be manipulated to hold therapeutic agents—including drugs, proteins or nucleic acids—and specifically target tumour cells. This can be an ideal therapeutic delivery system because it may limit necessary systemic exposure and toxicities of traditional chemotherapy while increasing efficacy. Specifically, if we are able to target the upstream pathways involved in HCC tumour progression (i.e., Wnt/β‐catenin, PI3K/Akt or MAPK) involved in cell proliferation and survival, we can discourage cancer growth and metastasis. We could be able to alter the endogenous exosomes to deliver siRNA or miRNA specifically targeting oncogenic transcripts—this is an emerging platform [17]. We can also conceive of exosomal DNA as a therapeutic opportunity for HCC. Exosomes contain double‐stranded DNA from both nuclear and mitochondrial sources and have the potential for therapeutic opportunity. For example, exosomal DNA could be engineered to carry CRISPR‐Cas9, allowing for gene editing or disruption of specific gene mutations for novel effects based on genetic differences influencing cancer development. This approach provides the possibility to be targeted with extremely high specificity to cancerous cells with little off‐target effects on healthy tissues. It has been shown that exosomal DNA can be delivered as an exosome to the tumour site where gene‐editing components could reduce the ability of the tumour to behave, at a genetic level [18].

Exosome‐based strategies can have many therapeutic applications in the treatment of HCC. They can be used as drug delivery systems and a basis for genetic therapy. These new methods of HCC treatment are a platform for developing improved treatment methods, with concurrent reduction in invasiveness and potential impact on the clinical management of this difficult cancer [19].

## 4 Exosomes as Therapeutic Agents

Exosomes are vesicles of endocytic origin that are secreted from any type of cell, including immune cells and cancer cells. They are classified according to their cellular origin and molecular cargo. In general, exosomes can be derived from mesenchymal stem cells (MSCs), immune cells (dendritic cells and macrophages) and specific cancer cells; any type may have an available therapeutic usage. Exosomes from MSCs have regenerative properties and provide some level of immune modulation, and they have been shown to be suitable for the treatment of inflammatory diseases and as a repair mechanism [20]. Exosomes from immune cells enhance vaccine immunogenicity, and exosomes from cancer cells contain tumour antigens and can also be utilised for antitumour therapies [21] (Table 1).

**TABLE 1:** Various sources and various types of exosomes, along with their specific mechanisms of action in HCC.

Source of exosomes	Type	Key components	Mechanism in HCC
MSCs	Regenerative Exosomes	Growth factors, cytokines, miRNAs	Promote tissue repair and regeneration; modulate immune responses to suppress tumorigenic inflammation
Immune cells	Immunomodulatory Exosomes	Cytokines, antigenic peptides, immune‐regulatory miRNAs	Enhance antitumour immune response by presenting tumour antigens and activating T cells
Hepatocytes	Hepatic Exosomes	Liver‐specific enzymes, liver function‐related miRNAs	Involved in liver homeostasis, potentially used for targeted delivery of drugs to liver tumour cells
HCC cells	Tumour‐derived Exosomes	Oncogenic proteins, mRNAs and miRNAs	Promote tumour growth, angiogenesis, and metastasis; could be used for vaccine development against HCC
Endothelial cells	Angiogenic Exosomes	Angiogenic factors, endothelial‐specific miRNAs	Promote angiogenesis within the tumour microenvironment, supporting tumour growth and metastasis

**FIGURE 5:**
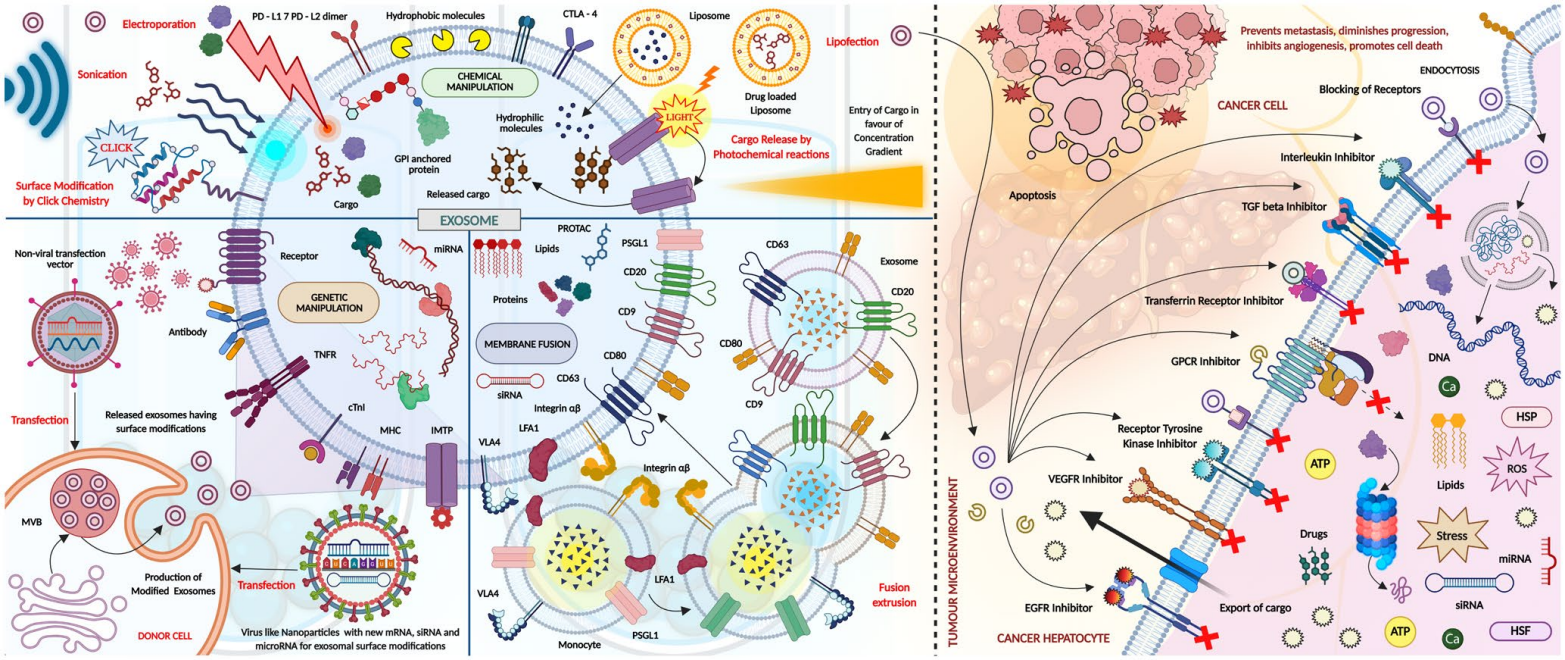
Engineered exosome for enhanced HCC targeting. Exosomes can be modified or engineered by different strategies (e.g., genetic manipulation, membrane fusion, surface functionalisation with drugs and anticancer peptides by Click Chemistry based approaches). These exosomes can be administered to the tumour site for enhanced targeting of HCC (Created in BioRender.com).

Exosomes can be engineered to improve their targeting and therapeutic efficacy. This can be done by modifying exosomes to express specific ligands that bind to receptors found on target cells to deliver cargo precisely. Modification of the surface proteins on exosomes relies on genetic engineering and protein engineering. For example, exosomes expressing the iRGD peptide, which binds integrins on the surface of tumour cells, were designed to specifically increase the targeted delivery of therapeutic molecules to the tumour microenvironment [22]. Another approach involves loading exosomes with therapeutic agents like chemotherapeutic drugs, siRNA or miRNA through electroporation, sonication or by exploiting the parent cell's biology to encapsulate these agents during exosome formation [23] (Figure 5).

**FIGURE 6:**
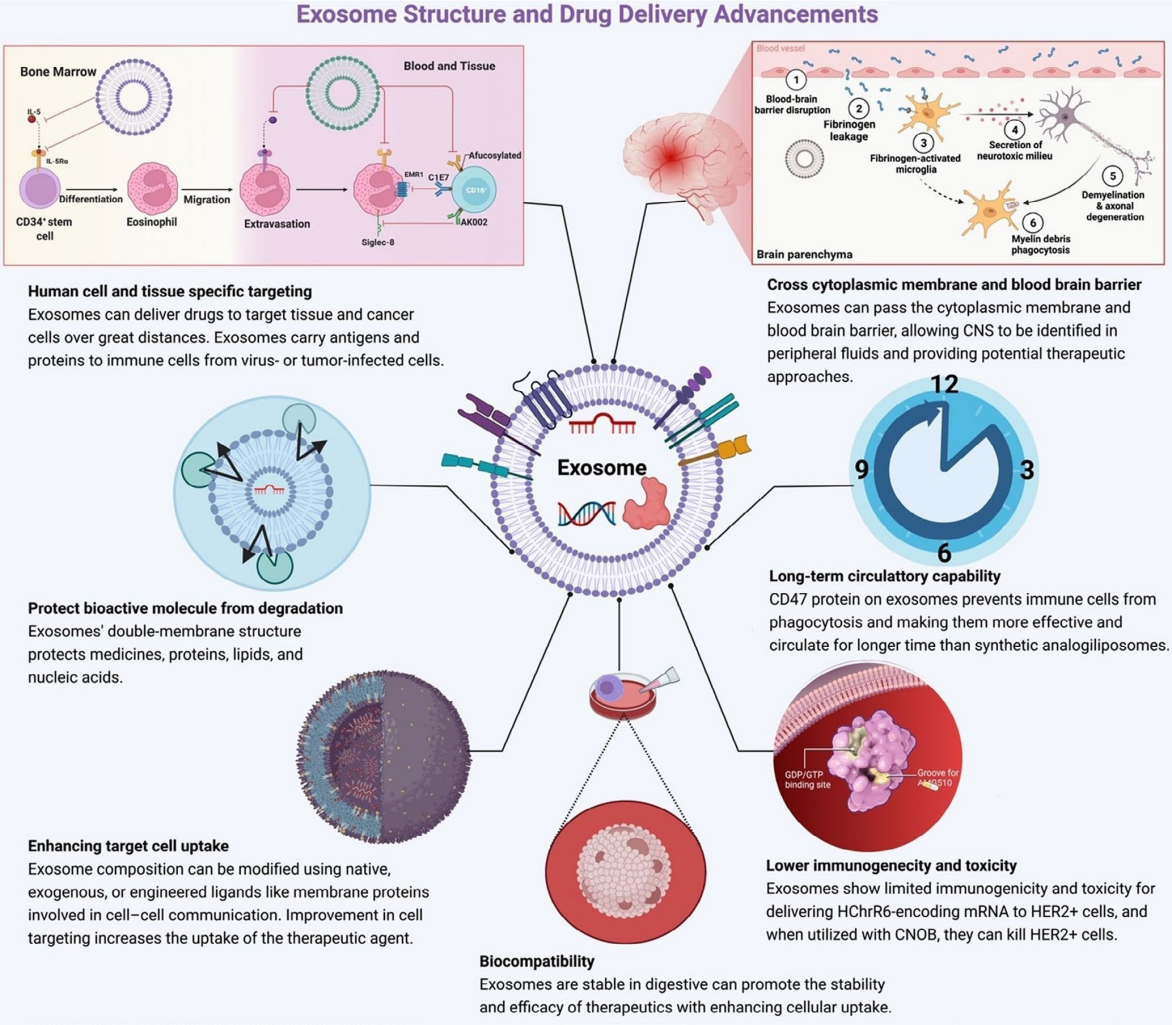
Exosome‐based drug delivery. (Reproduced with permission under Creative Commons CC BY 4.0 licence from ref. [8] Copyright 2022 The Authors).

Exosome‐based delivery systems offer numerous advantages over traditional drug delivery mechanisms. Their biocompatibility stems from their endogenous origin, which significantly reduces the risk of adverse immune reactions. Furthermore, their ability to cross biological barriers, such as the blood–brain barrier, makes them ideal candidates for treating diseases like brain tumours or neurodegenerative disorders [24]. Exosomes also possess an inherent targeting ability due to their surface molecules, which can naturally home to specific tissues or be engineered to enhance this specificity. Additionally, the small size and robust nature of the exosomal membrane allow for a prolonged circulation time in the bloodstream, increasing the likelihood of the exosomes reaching their target [25] (Figure 6).

## 5 Diagnosis and Prognosis of HCC With Exosomes

HCC diagnosis and prognosis are critically dependent on exosomes, functioning as vehicles for various forms of RNA and proteins to be delivered. These nanometre‐sized vesicles are powerful mediators of intercellular communication that significantly affect the tumour microenvironment, affecting growth, spread and interactions with the immune system. Recent studies have shown that exosomal biomarkers are both corelatable with disease and are capable of providing prognostic information for both disease and treatment response, making them easily accessible targets for early disease detection and monitoring with biofluids such as serum and plasma. Researchers showed that exosomal miRNAs and lncRNAs are abundant and have substantial diagnostic and prognostic implications for HCC, like miR‐122, miR‐125b, miR‐145 and miR‐192, and others are identified in serum indicating HCC and potentially differentiating it from liver cirrhosis or providing early diagnostic clues [26, 27]. In addition, TP53 mutations in exosomal DNA have been related to poor prognosis and poor recurrence‐free survival [28]. Additionally, particular exosomal biomarkers are related to tumour metastasis and invasion. Both miR‐18a, miR‐27a and miR‐20b type miRNAs in plasma serve as biomarkers for metastasis and prognosis because of the key role of exosomes in cancer pathophysiology [29, 30]. Exosomes have potential utility in therapeutic realms such as delivery vehicles for targeted therapies to improve therapeutic response [31] and for delivering treatments to providers for assessment of treatment response [32].

Table 2 elucidates various candidate exosomal biomarkers, their associated biofluids, potential mechanisms and their clinical significance for HCC. For instance, a combination of microRNAs like miR‐122, 125b, miR‐145, miR‐192, miR‐194, miR‐29a, miR‐17‐5p and miR‐106a in serum is highlighted as promoting cancer cell proliferation and invasion, thus serving as biomarkers for HCC diagnosis. Another example is miR‐106a, solely in serum, which also promotes cancer cell proliferation and is considered a biomarker for poor prognosis. Moreover, miR‐370‐3p and miR‐196a‐5p, also found in serum, are noted for promoting both cell proliferation and migration, indicating their significance in both diagnosing HCC and predicting poor prognosis. Each biomarker and its mechanism play a crucial role in understanding and managing HCC, offering insights into potential therapeutic targets and diagnostic markers [12].

**TABLE 2:** Exosomal biomarkers, their associated biofluids, potential mechanisms of action, and clinical significance for HCC diagnosis and prognosis [12].

Candidate exosomal biomarkers	Biofluid	Potential mechanisms	Potential clinical significance for HCC
miR‐122, 125b, miR‐145, miR‐192, miR‐194, miR‐29a, miR‐17‐5p and miR‐106a	Serum	Promotes cancer cell proliferation and invasion	Biomarkers for diagnosis
miR‐106a	Serum	Promotes cancer cell proliferation	Biomarker for poor prognosis
miR‐370‐3p, miR‐196a‐5p	Serum	Promotes cancer cell proliferation, invasion and migration	Biomarkers for diagnosis and poor prognosis
Combo of miR‐122, miR‐148a and AFP	Serum	Distinguishes early HCC from liver cirrhosis	Distinguishing early HCC from liver cirrhosis
Combo of miR‐10b‐5p, miR‐221‐3p and miR‐223‐3p	Serum	Distinguishes low AFP‐HCC	Distinguishing low AFP‐HCC

## 6 Exosomes in Hepatocellular Carcinoma

Exosomes play a multifaceted role in the pathogenesis of HCC, acting as key mediators in the communication between tumour cells and their microenvironment. These vesicles facilitate various aspects of tumorigenesis, including angiogenesis, immune modulation and the reprogramming of metabolic pathways in the liver. Exosomes released by HCC cells carry oncogenic proteins and nucleic acids that can transform nontumorigenic cells into tumorigenic forms, promoting cellular proliferation and sustaining malignant growth [33]. Additionally, exosomal miRNAs from HCC cells can downregulate tumour suppressor genes in recipient cells, further enhancing tumour growth and survival [34]. These mechanisms illustrate how exosomes contribute directly to the molecular and cellular changes required for HCC initiation and progression.

The potential of exosomes as biomarkers for early detection of HCC is significant, given their presence in easily accessible body fluids such as blood and urine and their cargo, which mirrors the molecular characteristics of the parent tumour cells (Table 3). Exosomes contain specific proteins and miRNAs associated with HCC, which can be detected before clinical symptoms appear, potentially leading to earlier diagnosis and treatment [43]. Elevated levels of specific exosomal proteins, such as AFP (alpha‐fetoprotein), which is commonly overexpressed in HCC, can serve as a diagnostic marker. Similarly, patterns of exosomal miRNAs have been identified that distinguish HCC from benign liver diseases and healthy controls, providing a noninvasive diagnostic tool that could supplement existing imaging and biopsy methods [44].

**TABLE 3:** Exosomal biomarkers for the early detection of HCC.

Biomarker type	Exosomal biomarker	Role in HCC	References
Proteins	AFP	Widely used serum marker, elevated in exosomes from HCC patients, indicative of tumour presence and burden	[35]
	Glypican‐3 (GPC3)	Overexpressed in HCC; detected in exosomes, potentially useful for diagnosing early‐stage HCC	[36]
	CD44	Associated with cancer stem cells; exosomal CD44 levels correlate with HCC aggressiveness and metastasis	[37]
miRNAs	miR‐21	Promotes oncogenesis; elevated levels in exosomes from HCC patients, linked to poor prognosis	[38]
	miR‐122	Liver‐specific miRNA; drastic reduction in circulating exosomal miRNA in HCC patients compared to healthy individuals	[39]
	miR‐223	Involved in cell proliferation and migration; increased levels in HCC exosomes, potential early marker	[40]
lncRNAs	HULC (Highly Upregulated in Liver Cancer)	Upregulated in HCC tissue and exosomes, serves as a possible marker for early detection	[41]
	MALAT1 (Metastasis Associated Lung Adenocarcinoma Transcript 1)	Involved in metastasis; higher exosomal levels found in HCC patients, indicative of progression and potential metastasis	[42]

Exosomes influence HCC progression and metastasis through multiple pathways. They can alter the tumour microenvironment by inducing angiogenesis and modulating immune responses, thereby creating conditions favourable for tumour growth and spread. Exosomal content such as VEGF promotes blood vessel formation, supplying nutrients to rapidly growing tumours [45]. Moreover, exosomes from HCC cells can prepare distant organs for metastasis by modifying the extracellular matrix and inducing a pre‐metastatic niche, a process supported by the transfer of metastasis‐associated proteins and miRNAs [46]. Exosomal integrins can dictate organotrophic metastasis, guiding HCC cells to specific organ sites, which enhance metastatic efficiency [47].

Exosomes utilisation in the HCC management is a major leap for oncology and is attributed to providing a string of noninvasive diagnostics along with innovative therapeutic strategies. Utilising its ability to mirror the molecular landscape of tumours as a window into the cellular mechanics of the cancer, they offer prospects for improved patient outcomes through tailored therapeutic approaches based upon what the cancer is doing. The prospect of exosomal research being integrated into clinical practice to revolutionise the management of HCC supports information that needs to continue to be explored in this exciting area of medical science.

## 7 Therapeutic Applications of Exosomes in HCC and Beyond

Exosome‐mediated drug delivery systems are a new and exciting advancement in therapeutics for HCC as they are biocompatible, have low immunogenicity and can encapsulate several therapeutic agents. One innovative approach in this area is microneedle‐assisted drug delivery, which improves the transdermal delivery of the exosome‐encapsulated drugs, a noninvasive and easy way to administer therapeutics directly to either systemic circulation or targeted tissue. Through microneedles, it can be possible for a patient to receive an amount of therapeutics conveniently while combining the targeting capabilities of the exosome and the high‐efficiency delivery of a microneedle. This combination would likely enhance drug bioavailability and bioefficacy for oncologic therapeutic modalities [35].

Much attention is being given to the use of exosomes as cargo for chemotherapeutics with the goal of reducing systemic toxicity known with conventional chemotherapy; exosomes can be loaded with chemotherapeutics such as doxorubicin or paclitaxel, they can preferentially target the HCC cells and not impact surrounding healthy tissue. In addition to targeting ability, exosomes can provide a therapeutic index that increases the utility of chemotherapeutics/antineoplastics while reducing the severity of side effects, improving patient quality of life and tailored outcomes [36]. Furthermore, exosome‐enclosed drugs have shown their efficacy remains high with a substantial potential for the inhibition of tumour growth in vivo [37].

The delivery of nucleic acid‐based therapies via exosomes is an exciting new way to treat HCC given their ability to delivery precise break or alter gene expression in tumour cells. Exosomes can be designed to delivery therapeutically active nucleic acids such as siRNA or miRNA or CRISPR‐CAS9 reagents that specifically target genes associated with tumour growth, tumour cell survival and resistance. The precision associated with this therapy allows the development of exosome‐based therapies for HCC to target important oncogenic pathways particularly alterations in the PI3K/Akt/mTOR pathway, that could lead to more effective clinical outcomes [38].

Exosomes can also play an important role in regulating the immunological environment of HCC. Exosomes can be engineered to deliver tumour specificity antigen and deliver costimulatory molecules that promote immune responses targeting HCC cells. Dendritic cell‐derived exosomes (DEXs) have been shown to promote antitumour immunity by presenting tumour antigens to T cells and promote cytotoxic T cell responses against HCC. This type of cancer treatment will not only target the tumour but promote an even immune system‐mediated response which can be beneficial for long‐lasting tumour control and prevention of tumour recurrence [39].

Clinical studies have only recently started to investigate the potential of therapy using exosome‐based approaches for HCC. Recently, a Phase I clinical trial to study the safety and activity of exosome encapsulated miRNA‐122 was conducted because of its importance to liver homeostasis and tumour suppression. The preliminary results showed the therapy was well tolerated and could have a meaningful activity against tumour markers in HCC [40]. Another ongoing study is looking at the use of those exosomes originated from mesenchymal stem cells with certain miRNAs, which are implicating HCC proliferation pathways with the intent of studying the therapeutic achievement in regard to tumour progression and patient survival [41].

## 8 Technological Advances in Exosome Therapy

**FIGURE 7:**
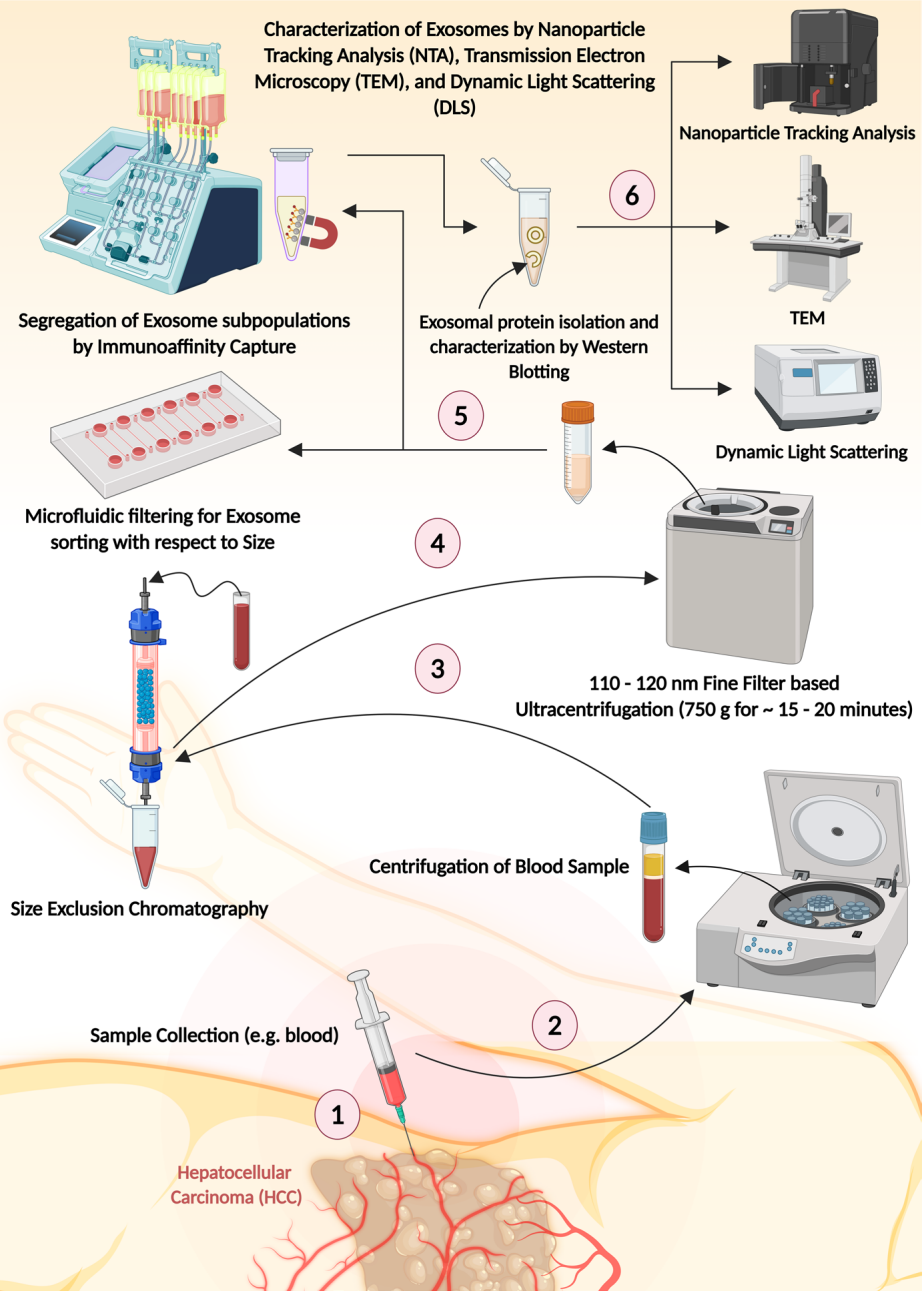
Exosome isolation and characterisation for advanced diagnostic and therapeutic applications (Created with BioRender.com).

The exosome research area has greatly evolved in terms of methods for isolation and characterisation, which has a significant importance for both investigating the biology and therapeutic purposes of exosomes (Figure 7). Conventional methods such as ultracentrifugation and density gradient centrifugation are frequently employed; however, the yield and purity are often low. In light of evolving technologies, methods such as size‐exclusion chromatography (SEC) and immunoaffinity capture (IAC) have been developed that provide enhanced purity and efficiency. Size‐exclusion chromatography, for example, separates exosomes by size, and studies have shown that it can maintain the integrity and biological activities of exosomes as compared to ultracentrifugation in isolation [42]. Immunoaffinity capture techniques utilise antibodies against typical exosomal markers, such as CD63 or CD9, and these methods are capable of isolating exosomes from complex biological fluids, which are indicative of diagnostic purposes [48].

Bioengineering of exosomes has allowed them to be utilised as exceptional vehicles for drug delivery with the capacity to target specific cell types or tissues. Exosomes can be genetically modified by inserting specific therapeutic genes, targeting ligands and even reporter genes into the exosomal membrane or cargo. An example of this is a study that engineered exosomes to express the rabies virus glycoprotein (RVG) peptide, which targets acetylcholine receptors, to deliver RNAi therapies specifically to the brain [49]. Alternatively, exosomes can be genetically modified to express surface proteins that specifically target and bind receptors that are overexpressed on cancer cells and achieve enhanced delivery of oncological therapeutics exclusively to the site of tumours [50].

**FIGURE 8:**
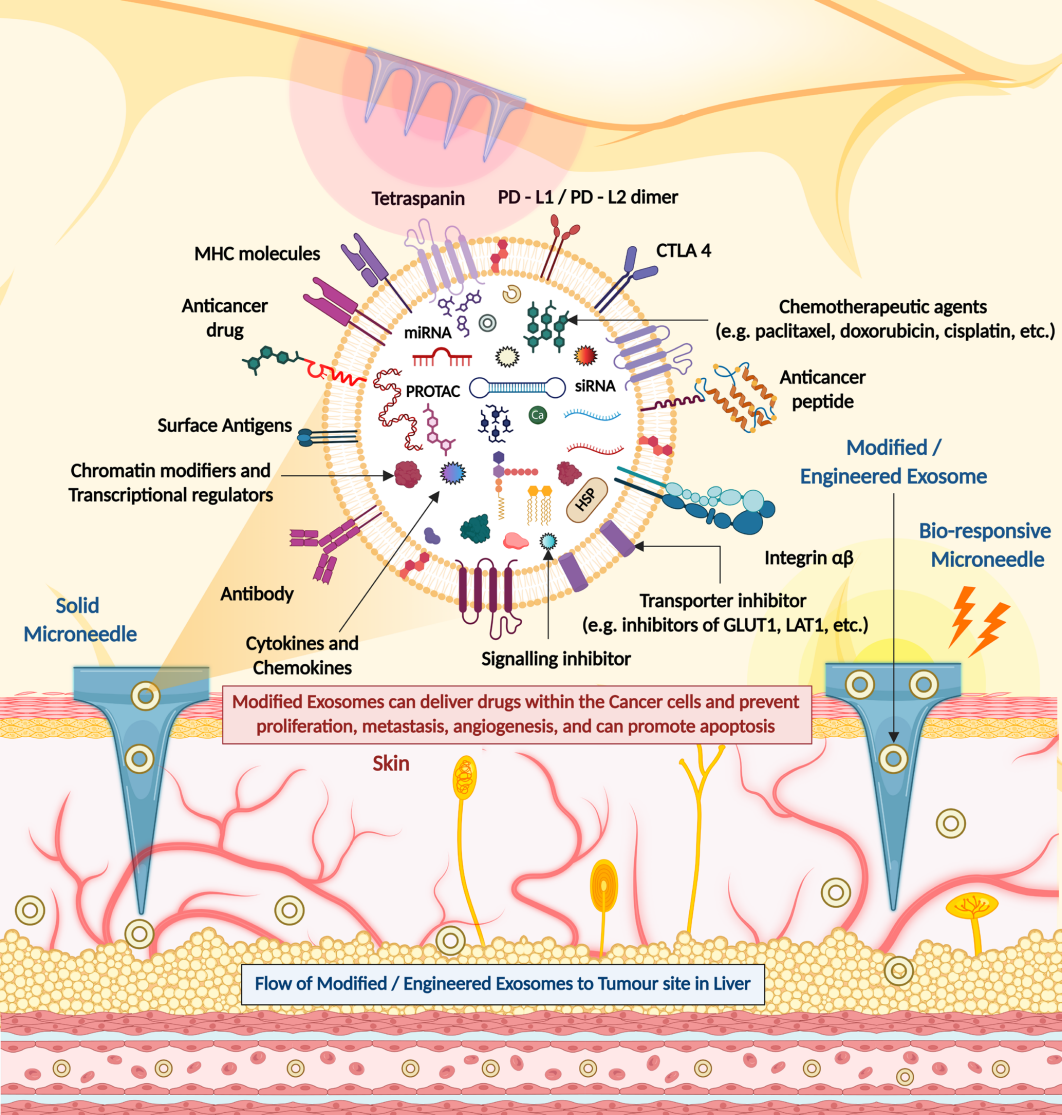
Microneedle assisted exosome‐based drug delivery in HCC (Created in BioRender.com).

The delivery systems for these exosome‐based therapies are also advancing, enhancing targeting specificity and therapeutic effects. One of the new options is hybrid systems, combining exosomes with nanoparticles or other delivery systems to reap the best of both delivery worlds. Exosomes have been coated with magnetic nanoparticles, allowing them to be directed to specific sites within the body using external magnetic fields [51]. This method significantly enhances the localised concentration of therapeutic agents, increasing the treatment's effectiveness while minimising side effects. Another method can be microneedle‐assisted exosomal drug delivery for more precise and targeted drug delivery, and this can reduce total off‐target effects (Figure 8).

Furthermore, the targeting mechanisms of exosomes can be enhanced by engineering their surface proteins to include specific ligands that recognise and bind to target tissues. This specificity not only increases the therapeutic payload's delivery efficiency but also reduces off‐target effects, crucial for treating diseases like cancer where precision is paramount [52].

## 9 Challenges and Limitations

A key obstacle to effectively treating HCC is the high degree of heterogeneity associated with such cancers. Heterogeneity can occur both between different tumours and within the same tumour in the same patient, in terms of their genetic, molecular and cellular profile and can impact the behaviour, progression and response to treatment, typically resulting in various therapeutic responses and challenges associated with managing disease. Exosomes, because of their natural role for cellular communication and ability to carry cargo, might present an innovative method of managing this heterogeneity [53]. Specifically, exosomes can potentially be used to create a more personalised treatment by providing specific therapeutic cargo that targets the various molecular pathways related to the distinct tumour subtypes. Exosomes can also be used to analyse the exosomal content derived from HCC patients in order to delineate specific biomarkers or molecular profiles representative of a tumour profile. This information could potentially allow researchers to develop exosome‐based therapies that are developed to target the specific tumour biology [54].

In addition, with exosomes as a delivery vehicle there is the added benefit of being able to package a range of therapeutic molecules such as siRNA, miRNA or components of CRISPR‐Cas9 that can be targeted toward oncogenic drivers found in different HCC subtypes. This not only enhances the specificity of the therapy, but can also improve the efficacy of the treatment by directing the therapeutic agent to its target cells, which would reduce off‐target effects and increase the therapeutic index overall [55].

Despite the promising trajectory of exosome therapy, there are still many technical and clinical hurdles that need to be addressed. Isolation and purification are multifaceted steps of the process of exosome delivery that employ complicated and expensive equipment and methods that can successfully delineate exosomes from other committing contaminants and vesicles. Properly standardising methods of isolation and purification is critical for establishing robust and reproducible exosomes for clinical application [56].

### 9.1 Exosomes Production Scalability Is Yet Another Major Barrier

To fulfil clinical efficacy, appropriate and inexpensive methods for the production of exosomes must be developed. Current production methods are labour‐intensive and do not produce enough quantities of exosomes for widespread clinical applications. Bioreactor technologies are an advanced technology that can scale up production, and genetic engineering may provide an opportunity to increase the yield of exosomes; however, this will require further research and validation [57]. In addition to the above‐mentioned considerations, there are regulatory hurdles necessary to account for as exosomes move toward being clinically relevant. Regulatory bodies such as the US Food and Drug Administration (FDA) require an enormous amount of testing and standardisation to enact the approval of an exosome‐based therapy treatment to ensure safety, efficacy and consistency [58]. Current frameworks for the regulatory oversight of emerging therapies are evolving, and guidelines need to be established for further development and transitional research of exosome therapies.

## 10 Looking Towards Future Development in Exosome Therapy

The future of exosome therapy is closely related to the continuous developments in exosome engineering to optimise their therapeutic benefits and broaden their applications. Innovations with exosome engineering include enhancing the targeting ability of exosomes mainly through surface modifications and genetic engineering. For example, exosomes are being engineered to express specific ligands that can only recognise and bind to specific receptors on cancer cells, which helps target delivery, providing the cargo specifically to the tumour microenvironment. This precision targeting reduces systemic side effects and improves therapeutic outcomes [59] (Figure 5).

Furthermore, there are advancements in the engineering of exosomes that escape the immune system, prolonging the circulation time and increasing their potential to deliver drugs or genes. Stealth exosomes are engineered by attaching polyethylene glycol (PEG) moieties to their surface to block the immune system recognising the exosomes as foreign [60]. Although there are enhancements for applications of exosomes in the treatment of cancer, these modifications have opened additional opportunities for use in multiple diseases in humans beyond cancer, such as neurodegenerative diseases and inflammatory diseases. As an exciting area of research, collaborative therapies could utilise exosomes with other treatment strategies such as chemotherapy, radiotherapy, or immunotherapy; for example, exosomes could be utilised to deliver chemosensitisers to tumour cells that have become resistant to standard chemotherapeutics to potentially reverse the resistance pathway and be more effective with the administration of chemotherapeutics [61]. Likewise, exosomes can be loaded with radiosensitisers to act on radioresistant tumour cells and be more effective with the endpoints of standard radiotherapy [62].

Exosomes are currently being evaluated as delivery agents for immune checkpoint inhibitors, which can activate the immune system against tumours, in the development of immunotherapy. By utilising exosome therapy and immune checkpoint blockade in tandem, this could give rise to enhanced tumour immunogenicity for immunotherapy, and ultimately improve the prognosis of immunotherapy [63, 64]. These strategies not only utilise the unique features of exosomes, but also synergise with what is already available, potentially resulting in greater clinical outcomes.

As developments in the field continue surveying clinical therapy predictions and trends, currently, a major prediction is the clinical forms of exosome‐based therapies being utilised in human health. The ongoing understanding of exosome biogenesis, payload manipulation and targeting suggests that exosomes will be arranged into precision medicine for personalised medicines based on patient profiles and disease characteristics [65, 66]. The rate of clinical trials developing in exosome therapies will increase alongside the greater focus on efficacy and safety across diseases. Furthermore, regulatory guidance is expected to keep pace with novel technologies that require regulated guidance; considering they are the technical solution to diseases, specific guidance and standards will be outlined for exosome production, characterisation and real‐world clinical usage [67].

## 11 Conclusion

In HCC, we have reached an exciting stage in the exploration of exosome‐based therapies and applications. Our current understanding emphasises the importance of exosomes in intercellular communication, particularly related to cancer‐specific developmental processes such as metastatic processes, immune evasion and drug resistance. Exosomes provide an opportunity to develop HCC therapies as a targeted drug delivery vehicle and a novel biomarker for early disease detection. For example, engineered exosomes are able to deliver chemotherapeutic agents and genetic material specifically to tumours, wherein therapies reach the malignant cells without affecting nonmalignant tissue [68]. In clinical use, exosomes are still in the early stage, with early‐phase clinical trials examining the potential for establishing exosomes as carriers of therapeutic payloads and as an adjunct in diagnostic processes in oncology.

The objective of these trials is to validate the safety, efficacy and delivery of exosome‐mediated therapies, although the results thus far have been encouraging for future developments and refinements [67]. There remain several important unanswered questions that will need to be investigated further. One especially important area involves methods of isolation and purification regarding their scalability and reproducibility, which will be essential for clinical purposes. Current methods need to be improved such that exosomes can be produced as: 1) sufficiently large quantities (given that a typical exosome dose can range from 10^10–10^12 exosomes) for clinical needs; and 2) exosomes are produced in sufficient purity that the product can be trusted at a clinical level [69]. There is also the question of the biological complexity and heterogeneity of exosomes, which makes it difficult to standardise therapeutic applications. This leads to several important questions that need further basic research in order to better understand how exosome cargo can be changed and manipulated to provide the best therapeutic outcomes and how exosomes interact with the immune system from patient populations with different characteristics [70]. Another important area includes the regulatory environment. As exosome‐based therapies move closer to being a part of standard care, regulatory guidelines will need to be established and refined to manage their development and ensure patient safety effectively. This includes defining clear parameters for manufacturing processes, quality control and ethical considerations, especially as the therapies involve manipulation at a cellular and molecular level [71]. Lastly, while the potential for exosomes to revolutionise cancer therapy is clear, the integration of these therapies into existing treatment paradigms requires careful planning. It involves understanding where they can be most effective within the standard treatment regimen and identifying which patients are most likely to benefit from such approaches [72, 73].

## Author Contributions

**Subham Sarkar:** conceptualization (equal), validation (equal), writing – original draft (equal), writing – review and editing (equal). **Ashok Kumar Bishoyi:** conceptualization (equal), validation (equal), writing – original draft (equal), writing – review and editing (equal). **R. Roopashree:** conceptualization (equal), validation (equal), writing – original draft (equal), writing – review and editing (equal). **Vishal Thakur:** conceptualization (equal), visualization (equal), writing – original draft (equal), writing – review and editing (equal). **Manpreet Kaur:** conceptualization (equal), validation (equal), writing – original draft (equal), writing – review and editing (equal). **Pusparghya Pal:** conceptualization (equal), validation (equal), writing – original draft (equal), writing – review and editing (equal). **Taha Alqahtani:** conceptualization (equal), validation (equal), writing – original draft (equal), writing – review and editing (equal). **Ali Alqahtani:** conceptualization (equal), validation (equal), writing – original draft (equal), writing – review and editing (equal). **Daniel Ejim Uti:** conceptualization (equal), validation (equal), writing – original draft (equal), writing – review and editing (equal). **Esther Ugo Alum:** conceptualization (equal), validation (equal), writing – original draft (equal), writing – review and editing (equal).

## Ethics Statement

The authors have nothing to report.

## Consent

The authors have nothing to report.

## Conflicts of Interest

The authors declare no conflicts of interest.

## Data Availability

All used data are within the manuscript.
